# Novel Higher-Order Spectral Cross-Correlation Technologies for Vibration Sensor-Based Diagnosis of Gearboxes

**DOI:** 10.3390/s20185131

**Published:** 2020-09-09

**Authors:** Len Gelman, Krzysztof Soliński, Andrew Ball

**Affiliations:** 1 Department of Engineering and Technology, The University of Huddersfield, Queensgate, Huddersfield HD1 3DH, UK; A.Ball@hud.ac.uk; 2 Meggitt Sensing Systems, Rte de Moncor 4, 1701 Fribourg, Switzerland; krzysztof.solinski@ch.meggitt.com

**Keywords:** wavelet spectral cross-correlation, gearbox fault diagnosis vibration analysis

## Abstract

Novel vibration sensor-based diagnostic technologies, built on the higher order wavelet spectral cross-correlation (WSC), are proposed, investigated and applied to gearbox vibration diagnosis for the first time in worldwide terms. The proposed WSC-based technologies do not feature any constrains in selection of signal spectral components, relations between which are analysed. That is a radical improvement in comparison with the higher-order spectra (HOS). The WSC technologies are applied for an experimental diagnosis of a local gear tooth fault of a helical gearbox that is developed during a long duration gearbox endurance test. Differences between the applied technologies and advantages of the novel WSC approach over the classical HOS are explained in detail. Superiority of the WSC technologies is justified by high validity comprehensive experimental comparison with the HOS technologies: i.e., the wavelet bicoherence and the wavelet tricoherence.

## 1. Introduction

Local gear faults generate short duration force impulses each time a contact with a damaged tooth surface takes place. Resulting transient vibrations have non-stationary, non-linear and non-Gaussian character and their low energy is distributed over multiple frequency ranges. Because of non-linearities in gears, fault-related vibration transients usually exhibit cross-correlation between different spectral components.

Various non-linear indicators are widely used for vibration sensor-based diagnosis and prognosis of machinery and structures. An important class of non-linear indicators for diagnosis and prognosis is based on the higher order spectra (HOS). These indicators may provide more insight into diagnostic capabilities of the vibration signal analysis. Fackrell, Collis, White and Hammond introduced a new approach for condition monitoring, based on the normalised HOS: i.e., the Fourier-based bicoherence and the tricoherence (later referred as the “classical HOS”), that are measuring a system non-linearity in normalised scale between 0 and 1 [1,2,3,4]. As all cumulants featuring orders greater than two have zero value for the Gaussian signal components, the HOS are particularly useful for early stage machinery fault diagnosis, especially, for low signal to noise ratio (SNR) [5,6].

In cases of vibration signals, acquired under non-stationary speed conditions, a useful approach is order tracking [7,8]. Even for very small speed fluctuations, it is beneficial to perform order tracking to make sure, that every signal section, corresponding to fundamental rotation period of machinery, consist of the same number of samples, before subsequent signal-processing operations are preformed [9,10,11]. This signal-processing technique allows an efficient extraction of spectral components that are integers or fractional multiples of the fundamental rotation frequency, which is usually linked to kinematics of the diagnosed objects, helps avoiding the influence of the spectral leakage and, therefore, improves the signal to noise ratio of analysed spectral components. In research, presented in this paper, gearbox is operated under the constant speed and load conditions. Although there are extremely low speed fluctuations, vibration signals were resampled; however, the spectral representation of signals is presented in absolute frequency domain.

A useful method for analysing non-linear and non-stationary data is the empirical mode decomposition, in which any complicated vibration data can be decomposed into a number of intrinsic mode functions that admit the Hilbert transform [12]. This method is widely used for bearing and gear diagnosis and prognosis. In [13], the ensemble empirical mode decomposition method is employed to demodulate the envelope of the bearing signals and the fault characteristic frequencies of the vibration signals were acquired. Based on this approach, the characteristic frequency ratio for fault identification is defined. In [14], gearbox fault diagnosis is performed by integrating ensemble empirical mode decomposition with the permutation entropy. In [15], variational mode decomposition, combining with noise-aided data analysis, are employed for fault diagnosis of a gearbox. In [16], the orthogonal empirical mode decomposition is exploited for in-service bearing fault diagnosis. However, this promising method is still the second order technique and, therefore, less effective for gear and bearing early fault diagnosis, than the HOS.

The superiority of the higher order spectra (HOS) over the second-order transforms for fault and non-linearity diagnosis at the early stage is well known and described in multiple works, e.g., [1,17,18,19,20], related to fault diagnosis by the HOS. The main reason of that superiority is that, unlike the traditional and novel second order techniques (e.g., the Fourier transform, the wavelet transform, the Wigner–Ville distribution, etc.), the HOS are employing simultaneously multiple spectral components, generated by a fault, in contrast to the second order techniques, that are employing single spectral components. Therefore, the HOS consider simultaneous interactions between these spectral components, e.g., the quadratic phase coupling for the bispectral analysis, etc. The simultaneous use of multiple spectral components and the simultaneous interactions between these components provide an advantage to the HOS, compared with the second-order techniques, in terms of early fault diagnosis. Another important advantage of the HOS, compared with the second-order techniques, is HOS’s ability to suppress noise in data [21] and, thus, to improve the effectiveness of early fault diagnosis in presence of noise. It is also well known and described in multiple classical works that the second-order wavelet transform is superior over other second order transforms in terms of effective diagnosis of transient impacts, generated by gearbox and bearing local faults. 

Taking into account the above two considerations, the combination of the HOS with the wavelet transform, i.e., the wavelet HOS [22,23,24,25,26,27], is superior over the classical HOS for early local fault diagnosis in gearboxes and bearings. The higher effectiveness of the wavelet HOS over the classical HOS is shown in the pioneering research work [22]. 

Finally, it is shown in the present work by the comprehensive experimental trials, that the proposed higher order spectral cross-correlations, based on the wavelet transform, are superior over the wavelet HOS for local fault diagnosis in gearboxes.

In summary, considering all the aforementioned considerations and comparisons, it is concluded here on superiority of the proposed technologies over all other fault diagnosis technologies for local fault diagnosis in gearboxes and bearings, based on transient impacts, generated by gearbox and bearing local faults.

HOS applications in the field of diagnostics are the subject of numerous research works and the HOS based approach is investigated for both stationary (e.g., the classical Fourier based HOS) and non-stationary signals (e.g., the short time chirp-Fourier based HOS). The HOS techniques developed thus far utilised 3rd and 4th order statistics, with the Fourier transform, the short time chirp-Fourier transform [28] or the wavelet transform [29], that are used as their kernel functions. 

The bispectrum and the trispectrum are used for vibration diagnosis of rolling element bearings in [30,31,32,33]. Because of the complex nature of results, given by the bispectrum and the trispectrum, number of multi-feature classification algorithms are used, including the k-nearest neighbours and neural network techniques, to diagnose damaged and undamaged bearings [34,35]. The bicoherence for gear and bearing fault diagnosis is applied to a helicopter gearbox [36]. This research is specifically aiming the diagnosis of signal changes at a priori known frequency pairs for certain bearings and gearbox faults that are manifesting harmonic patterns. The classical bispectrum is also applied for damage diagnosis for industrial gearboxes [37,38,39]. The bicoherence is applied for vibration fatigue crack diagnosis in [40]. In this research, two bicoherence methods are compared. The first traditional method is based on the bicoherence magnitude, whereas the second novel method utilized the real and the imaginary parts of the complex bicoherence. It is shown that usage of the real and the imaginary parts of the bicoherence essentially improves the certainty of the diagnosis [40]. Later on, optimisation of bicoherence parameters for monitoring fatigue and accompanying changes of mechanical system stiffness are investigated in [41]. The bicoherence of the enveloped vibration signal is applied to fault diagnosis for rolling element bearings [42].

In order to adapt the HOS approach to analysis of signal components, featuring variations of the instantaneous frequency, HOS techniques, based on the short time chirp Fourier transform [43] and the chirp Wigner transform [44,45], are proposed. The higher order chirp Fourier approach is successfully used for detection of instabilities in a gas turbine combustor [28]. The bispectrum is broadly used diagnostic oriented signal analysis. The bispectrum-based approach is used for modulation signal analysis of vibration signals of the spur gearbox in order to evaluate the gearbox condition [46]. The bispectrum of vibration signal modulation is applied for planetary gearbox monitoring [47].

All these works are related to the usage of multiple spectral narrowband components, generated by damage, for HOS estimation and damage diagnosis. Although efficient in detecting small changes buried deep in the broadband noise, these methods did not offer the capability of fault localisation. Therefore, further investigation on application of the HOS for damage diagnosis for rolling element bearings and gearboxes resulted in the development of a new approach for gear and bearing diagnosis, that is not employing multiple narrowband components, generated by a damage.

In a new approach, presented in this paper, higher-order techniques are utilising multiple spectral components, that are, normally, not narrowband, and contained in multiple frequency bands, where transient vibrations, related to machinery faults, are present.

This approach employs the wavelet transform as a HOS kernel function, which adds capability of signal analysis in both time and frequency domains. Time dimension can be easy associated to specific angular position mechanical element what enables fault localisation. The wavelet transform as a HOS kernel function is first introduced in 1995: i.e., a locally averaged wavelet bicoherence as a tool for turbulence analysis [22]. However, the locally averaged wavelet bicoherence is not suitable to signals, which are not locally stationary. Therefore, the novel instantaneous wavelet bicoherence is proposed in [23] for non-linear and non-Gaussian transient signal components. In this approach, the wavelet transform, which is the base for the instantaneous wavelet bicocherence (WB) estimation, is evaluated for transient gear and bearing signals. The instantaneous wavelet bicoherence approach is employed for locomotive roller bearings diagnostics [24].

The choice of frequency bands for WB features is determined by evaluation of the wavelet scalograms of signals, originating from damaged gears and bearings. It is critical to choose relevant frequency bands for WB diagnostic features to ensure reliable fault detection. Also, knowledge of relevant frequency bands before the WB features are estimated, has significant influence on optimizing WB calculation time. The locally averaged and instantaneous WB-based technologies are applied to rolling element bearings fault detection and localisation [26,34,48]. The WB is applied for detection of pitting on a gearbox in [27]. For the proposed approach, the selection of frequency bands is based on evaluation of the averaged WB modulus maps.

From the definition of the HOS, it is clear that all the analysed spectral components must exhibit amplitude and phase coupling in order to obtain a non-zero result that is associated with a mechanical system fault. HOS techniques, the 3rd-order bispectrum and 4th-order trispectrum, and their normalised versions, the bicoherence and the tricoherence, by definition require a strict choice of spectral components. Specifically, the highest frequency, taken into consideration, has to be a sum of remaining lower frequencies: i.e., in the case of the 3rd-order bispectrum it is (*f*_1_ + *f*_2_), and in the case of the 4th-order trispectrum it is (*f*_1_ + *f*_2_ + *f*_3_) [1].

This requirement will be fulfilled in some cases, in which machinery faults generate spectral components, that are presenting an explicit harmonic pattern. In cases of machinery faults, generating a transient vibration signal, energy of which is broadly distributed over the frequency domain, the HOS requirement may not be met for all spectral components, that are carrying diagnostically relevant information. Therefore, this HOS requirement is limited spectral components, that could be used for diagnostics and, thus, limited damage types, that could be effectively detected by the classical HOS.

Another disadvantage of the HOS technologies is that the normalized HOS of order *n* do not represent the cross-correlation between *n* complex spectral components, related to faults in electro-mechanical systems. 

In order to mitigate these constraints, the proposal here is novel higher-order spectral vibration sensor-based diagnosis technologies, built on the spectral cross-correlation, that could take into account statistical dependencies between all needed combinations of multiple spectral components, appearing due to gearbox damage.

Other novelties of this research are as follows:

- novel experimental validation of the proposed technologies via comprehensive experimental trials;
- novel experimental comparison of the proposed technologies with higher-order spectra technologies;
- novel experimental comparison of the proposed spectral cross-correlation technologies of order 3 and 4;
- novel method of identification of diagnostically relevant frequency bands for the proposed technologies.

The proposed technologies are validated by comprehensive experimental trials. Novel comparison between the wavelet spectral cross-correlation (WSC) of 3rd and 4th orders and corresponding classical HOS technologies is presented. New method of identification of diagnostically relevant frequency bands for the proposed technologies is introduced and explained in detail.

The main aim of the presented research is to propose and investigate novel vibration diagnosis technologies, based on higher-order WSC analysis of vibration signals. 

The objectives of the research are to:

- develop and investigate for the first time in worldwide terms novel vibration sensor-based diagnosis technologies, built on the higher-order wavelet spectral cross-correlation for gearbox vibration health monitoring;
- perform a novel experimental validation of the proposed technologies via comprehensive experimental trials;
- perform a novel experimental comparison of the proposed technologies with higher order spectra technologies;
- perform a novel experimental comparison of the proposed technologies of order 3 and 4.

## 2. The Higher-Order Wavelet Spectral Cross-Correlation

The higher-order wavelet spectral cross-correlation (WSC) of complex spectral components of order *n* is expressed by following Equation:

(1) $$\begin{array}{cc} & WSCn\;\left(f_{1},f_{2},\ldots ,f_{n},t\right)\\ & =\;\frac{E\;\left\{\;\left[W_{\Psi \;}\left(f_{1},t\right)\;-\;\bar{W_{\Psi \;}\left(f_{1},t\right)}\right]\;\left[\;W_{\Psi }\;\left(f_{2},t\right)\;-\;\bar{W_{\Psi \;}\left(f_{2},t\right)}\;\right]\ldots \left[\;W_{\Psi \;}\left(f_{n},t\right)\;-\;\bar{W_{\Psi }\;\left(f_{n},t\right)}\;\right]{\;}^{*}\;\right\}}{\sqrt{var\;\left(W_{\Psi \;}\;\left(f_{1},t\right)\;\right)\;var\;\left(\;W_{\Psi \;}\left(f_{2},t\right)\;\right)\ldots var\;\left(W_{\Psi }\;\left(f_{n},t\right)\;\right)}} \end{array}$$

where *f*_1_, *f*_2_, …, *f_n_* are the frequencies, for which the *WSCn* is estimated, *E* is the mean value operator, ∗ denotes the complex conjugation, *var* is the variance operator, $\bar{W_{\Psi }}\;$ is the mean value of transform *W*_Ψ_, which is the continuous wavelet transform of the analysed signal *x*(*t*), given by:

(2) $$W_{\Psi }\;\left(a,t\right)\;=\;\frac{1}{\sqrt{\left|a\right|}}\overset{+\infty }{\underset{-\infty }{\int }}x\;\left(t^{\prime }\right){\;\Psi }^{*}\;\left(\frac{t^{\prime }-t}{a}\right)\;dt^{\prime }$$

where *a* is a scale parameter, that is inversely proportional to frequency of the wavelet transform, *t* is a time shift parameter and Ψ denotes a complex mother wavelet function.

The higher-order spectral cross-correlation (1) is a complex function, estimated by the wavelet transforms of vibration signals at *n* frequencies and depends on *n* frequencies. The significance of the wavelet spectral cross-correlation is that it is a measure of the statistical dependencies between multiple complex frequency components. 

The clear physical sense of the proposed technologies is that complex frequency components, that have appeared in the spectrum of measured vibration data due to gearbox local damage, exhibit non-zero spectral cross-correlation. Therefore, the *WSCn* is sensitive to the appearance of damage-related vibration spectral components. The normalization of the cross-correlation by the variances allows avoiding a misleading interpretation of the cross-correlations due to intensity changes of gearbox vibration data.

The spectral cross-correlations differ from the classical HOS. The normalised HOS of order n do not represent the cross-correlation between *n* complex spectral components.

If the proposed technologies are being applied to diagnosis of gearboxes, then interval for the WSC is (0–1). WSC magnitude values closed to 0 (i.e., low cross-correlation between spectral components) are related to the no damage case, while magnitude values closed to unity (i.e., a high cross-correlation between spectral components) are related to damage.

The integrated feature *IWSCn* is proposed here as the *WSCn* diagnostic feature. It can be analysed in time/angle domain; that allows an efficient fault localisation. The integrated wavelet spectral cross-correlation feature *IWSCn* is given by the following Equation:

(3) $$IWSCn\;\left(t\right)\;=\;\frac{1}{B_{1}B_{2}\ldots B_{n}}\int_{B1}\int_{B2}\ldots \int_{Bn}{\left|WSCn\;\left(f_{1},f_{2},\ldots ,f_{n},t\right)\;\right|}^{2}df_{1}df_{2}\ldots df_{n}$$

where *B*_1_, *B*_2_, …, *B_n_* denote widths of the integration frequency bands, in which spectral components at frequencies *f*_1_, *f*_2_,… *f_n_*, are located, |…| is the absolute value operator.

The integrated feature can be estimated for specific spectral components, contained in different frequency bands. 

In the present research, the WSC of orders 3 and 4 are employed: i.e., the IWSC3 and the IWSC4, expressed by the following Equations:

(4) $$\mathrm{IWSC}3\;\left(t\right)\;=\;\frac{1}{B_{1}B_{2}B_{3}}\int_{B1}\int_{B2}\int_{B3}{\left|\mathrm{WSC}4\;\left(f_{1},f_{2},f_{3},t\right)\;\right|}^{2}df_{1}df_{2}df_{3}$$

(5) $$\mathrm{IWSC}4\;\left(t\right)\;=\;\frac{1}{B_{1}B_{2}B_{3}B_{4}}\int_{B1}\int_{B2}\int_{B3}\int_{B4}{\left|\mathrm{WSC}4\;\left(f_{1},f_{2},f_{3},f_{4},t\right)\;\right|}^{2}df_{1}df_{2}df_{3}df_{4}$$

The integrated feature IWSC3 should be estimated for different spectral components triplets, belonging to one of the frequency bands, that are identified as diagnostically relevant. The IWSC4 feature should be estimated for different quadruple combinations of spectral components, belonging to one of the frequency bands, that are identified as diagnostically relevant. 

The IWSC3 and the IWSC4 contain information about statistical relations between specific spectral components, no matter their frequency domain location. That enables usage of short duration transient frequency components, energy of which is low and broadly distributed across frequency domain. IWSC3 and IWSC4 values, obtained for vibration signals, that are recorded for faulty gearbox, may indicate angular locations of pinion faults and be the base of reliable gearbox health monitoring.

**Figure 1:**
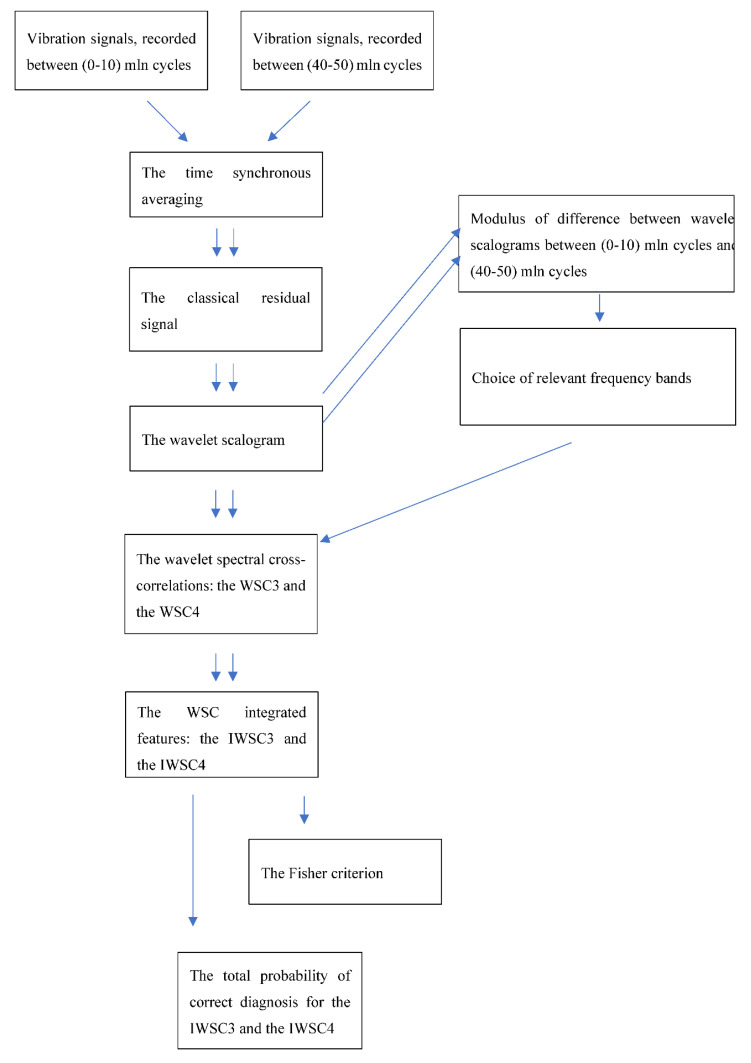
Flow chart of the wavelet spectral cross-correlation (WSC)-based signal processing technologies for gearbox diagnosis.

The block diagram below (Figure 1) shows the flow chart of digital signal processing steps, undertaken to apply the WSC to gearbox vibration data, including comparison of the obtained results via the Fisher criterion in order to identify angular locations of the damaged teeth. The total probability of correct diagnosis and the Fisher criterion are estimated in order to evaluate diagnostic effectiveness of the proposed technologies and to compare diagnostic effectiveness of the WSC3 and the WSC4 technologies.

## 3. Application of the Higher-Order Wavelet Spectral Cross-Correlations for Gearbox Damage Diagnosis

### 3.1. Test Rig Experimental Set-Up

**Figure 2:**
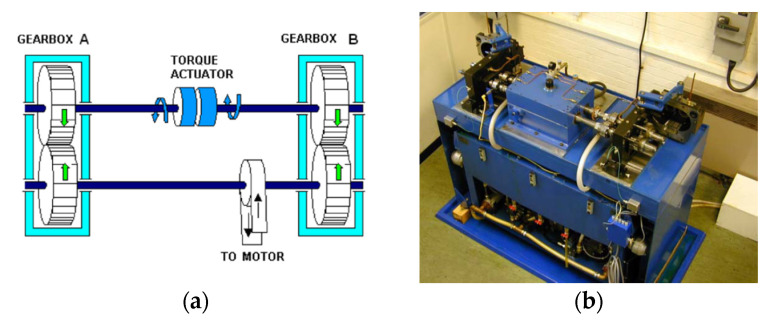
Scheme (**a**) and picture (**b**) of the gearbox test rig.

Analysed vibration sensor-based signals have been acquired during the overloaded gearbox endurance test, in which the natural pitting progression of the gear tooth was observed, resulting in tooth micro pitting. The object of the test was a 91.5 mm back-to-back gearbox test rig, provided by the Design Unit—Gear Research Centre of the University of Newcastle. The test rig (Figure 2) enables tests of gears, featuring module from 2 to 6 mm, face width up to 30 mm, helix angle up to 30°, under torque up to 1400 N and using a motor of 30 kW. Gears can be tested at a rotation speed up to 6000 rpm.

**Figure 3:**
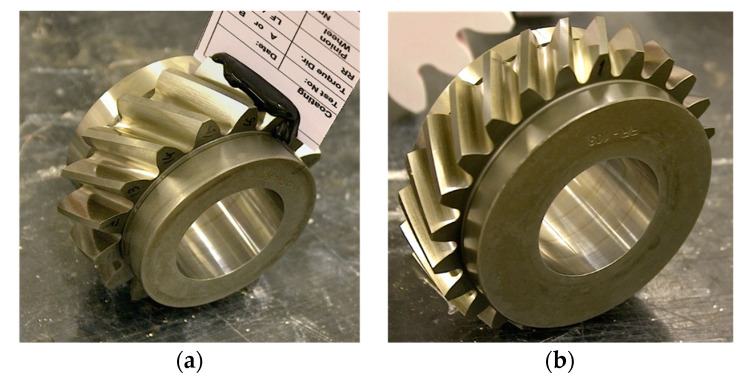
16 teeth pinon (**a**) and 24-teeth gear (**b**).

The test rig consisted of two gearboxes, featuring the same centre distance and ratio (16-teeth pinion, 24-teeth wheel). Two gearboxes were connected with torsionally compliant shafts. One of the shafts featured an interposed servo-hydraulic actuator, which allowed precise control of a loading torque during the test rig operation. The 3.9 mm module helical gears under test featured 25 mm face width, 30° helix angle and were made of carburised and tempered S156 steel (Figure 3). The pictures were taken before the start of the test.

The gearbox and the test rig allowed unique fully controlled experimental trials with a possibility to create a very early stage of natural pitting development (i.e., natural micro-pitting) and accurate stereo optical evaluation of pitting progression during the gearbox endurance test. This enabled comprehensive validation of WSC-based technologies. The main advantage of the proposed technologies is effective diagnosis of early stage of gear local tooth faults. Therefore, the gearbox and the test rig allowed us to comprehensively validate that important advantage. A very early stage of tooth local fault diagnosis is very important for many critical gear applications, e.g., aerospace, renewable energy, automotive, etc.

The analysed vibration signals and shaft speed data were recorded for the gearbox A in the axial direction using gear surface mounted, piezoelectric, positive polarity, charge, shear mode, linear accelerometer, type KCF AG107M, featuring sensitivity of 50 pC/g, transverse sensitivity below 5%, flat frequency response in the frequency range (0.5–6000) Hz (±1 dB), 80 g maximum measured acceleration and mass of 28 g. Piezo material of the accelerometer was PZT-5, isolation resistance was more than 10 × 10^9^ Ohms, capacitance was 1200 pF, temperature range was −40 to +150 deg C, shock limit was 800 g, temperature sensitivity is 4 mg/deg C, structural strain sensitivity was 0.2 mg/micro strain and magnetic field sensitivity was 2 g/T. The output connector featured water proof sealing. 

**Figure 4:**
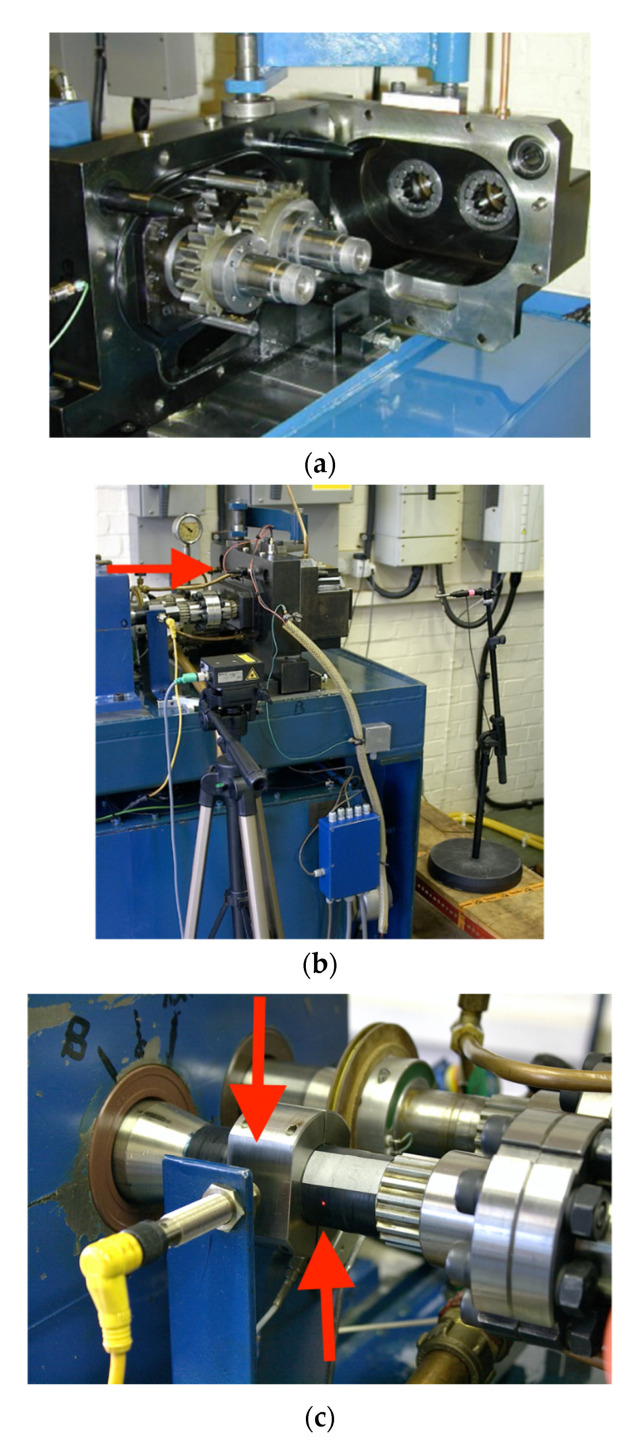

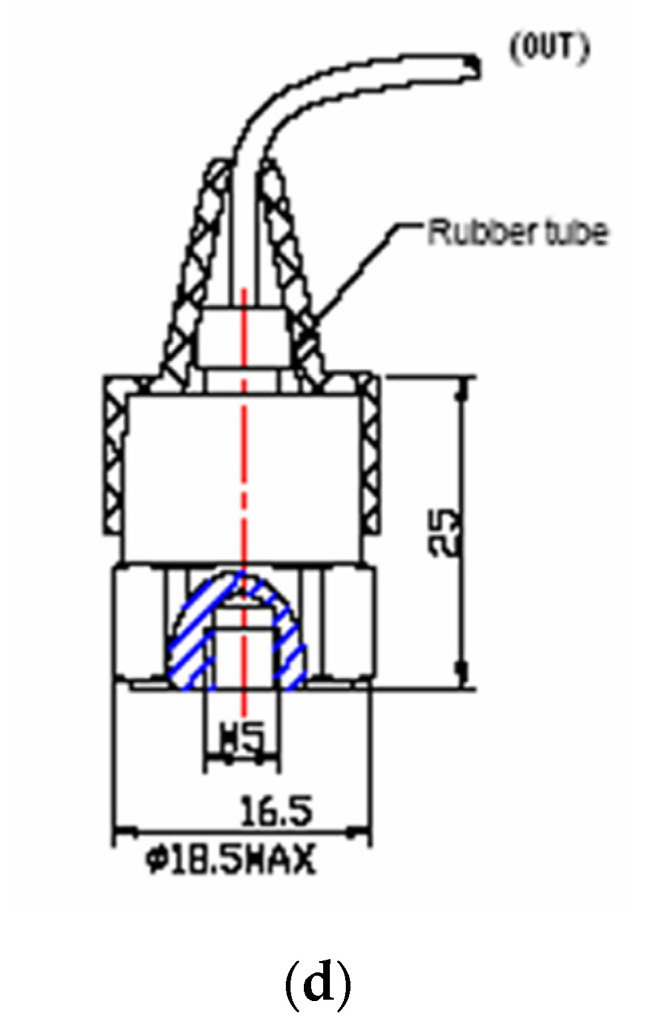
(**a**) Open gearbox (archive picture), (**b**) the assembled gearbox, (**c**) installation of eddy current and laser speed sensor. (**d**) Cross-section of the accelerometer.

The acceleration sensor was installed on the external housing of the gearbox in a location, that is close to the area, at which the gear and the pinion are meshing (Figure 4a,b) in order to minimise the overall transmission path effect. In Figure 4b, the accelerometer location is shown by a red arrow. 

The rigid accelerometer installation was made directly to the gearbox housing, using the standard removable stud of the accelerometer (stud diameter is M5), that is shown in Figure 4d of the accelerometer schematic, and a drilled and tapped hole on the mounting area of the external gearbox housing. The accelerometer mounting area was free from oil and grease, smooth, unpainted, flat and much larger than the base of the accelerometer.

Vibration acceleration and tacho signals (eddy current and laser speed sensors are shown in Figure 4c by two red arrows) were recorded with a sampling rate of 40 kHz. Due to frequency response characteristics of the accelerometer (the resonance frequency was 15 kHz), vibration signals were subjected to low-pass antialiasing filtering at a corner cut off frequency of 13.5 kHz, attenuation of the filter in the transition band is 80 dB/oct. During the test, the pinion was spinning at 3000 rpm (50 Hz), under a load of (500 ± 5) Nm. In total, the pinion performed 50 × 10^6^ cycles during the duration of the test.

During the test, a natural micro-pitting of tooth flank surfaces took place. Micro-pitting is a classical fatigue failure mode and results in a loss of material and, therefore, this fault has a very negative influence on gears geometry, as it creates destructive gear wear. As the liberated material wears away from the gear surface, the tooth shape and flank surface change; that causes a concentration a gear load over a smaller area, an oil film disruption, a reduction of gear accuracy, an increase of local tooth stress and, therefore, enables conditions for micro-pitting propagation. This would eventually have an impact on the accuracy of the gear mesh, resulting in decreased gear efficiency and reliability and essentially increased gear misalignment, noise and vibration. The development of micro-pitting is concerning and very undesirable as micro-pitting has the potential to very rapid growth and progress into another gear failure mode, such as a macro-pitting, that may lead relatively quickly to gear severe destruction and collapse. So, micro-pitting fault potentially leads to a decrease of gearbox reliability, safety and efficiency of operation. Therefore, diagnosis of a dangerous gear fault, micro-pitting, at very early stage, is very important for many critical gearbox applications, such as aerospace, renewable energy, automotive, including racing cars, etc.

After every 10^6^ cycles, the test rig was stopped and condition of the teeth surfaces were examined/diagnosed by optical stereo images of the pinion teeth flank surface replicas, in order to evaluate the progress of teeth natural micro- pitting and every single tooth, on which pitting was initiated, was correctly diagnosed. After pitting evaluation, the gears were mounted back on the rig in the same angular position.

**Figure 5:**
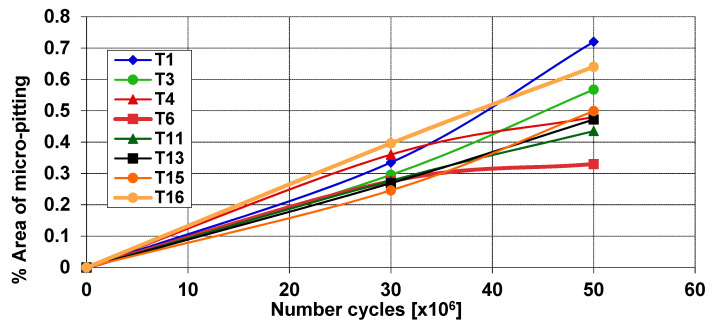
Estimation of progressing micro-pitting on the selected teeth of the pinion.

The plot below (Figure 5) shows the estimation of the micro-pitting progress on the pinion teeth, which showed damage [27].

**Figure 6:**
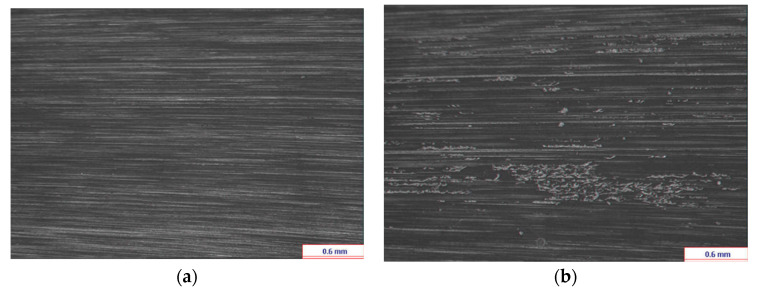
(**a**) Pinion tooth surface at the beginning of the experiment. (**b**) Pinion tooth surface at the end of the experiment, after 50 × 10^6^ cycles (**b**).

In the presented research, out of all signals available, five consecutive randomly selected files, which were recorded between (0–10 × 10^6^) cycles, represent the undamaged case, and five consecutive randomly selected files, which were recorded between (40 × 10^6^–50 × 10^6^) cycles, represent the case, where local gear faults were detected. For tooth 1, pinion tooth surface at the beginning of the experiment and pinion tooth surface at the end of the experiment, after 50 × 10^6^ cycles, are shown in Figure 6; the relative micro-pitting size of tooth 1 at the end of the experiment is 0.7% (Figure 6b). 

The pinion and gear featured helical teeth and the contact ratio of this gear pair was 2.4; that means that 40% of the time three out of 16 pinion teeth were in contact with the teeth of the gear. As a consequence, attribution of angular position of a fault (detected by vibration signal analysis) to the particular tooth was not possible and one angular position can be always attributed to two or three teeth. Therefore, in most of plots, that are featuring angular dimension, the angular ranges, in which particular teeth of the pinion were in contact with teeth of the gear, were marked with blue and red vertical lines and pinion teeth numbers.

**Figure 7:**
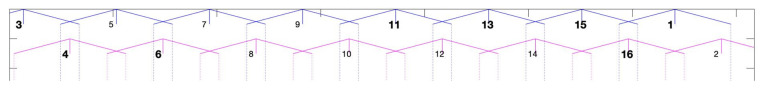
Marking of angular ranges, in which pinion teeth were in contact with gear teeth.

Numbers of teeth 1, 3, 4, 6, 11, 13, 15 and 16, on surface of which pitting was found, were marked with bold font (Figure 7). Such marking of angular ranges helps to compare results of analysis, presented in this paper, with results of assessment of surface pitting.

It is worth mentioning that, in a correctly designed gearbox, the contact ratio shall be greater than 1.2. For the gears, that are supposed to operate in quite manner, contact ratio shall be greater, than 2. Therefore, in practice, gearbox vibration signals rarely originate from a contact of single pair of meshing teeth and full separation between neighbouring teeth is not always possible.

### 3.2. Digital Signal Processing of Gearbox Vibration Data

#### 3.2.1. The Angular Signal Resampling, the Time Synchronous Averaging and Classical Residual Signal Estimation

The signal sections corresponding to one revolution of pinon, length of which was oscillating closely around 800 samples, were up sampled to 1024 samples per one revolution. Therefore, every sample of the signal could be associated with exact angular position of a pinion, what is necessary for locating the pinion teeth faults.

The resampled signal has been subjected to the time synchronous averaging (TSA), which suppresses signal components, that are non-synchronous to the rotating frequency of the pinion. Only the signal components that periodically appear with each pinion revolution are preserved. The TSA signal was estimated for 50 consecutive up sampled signal sections, corresponding to one pinion revolution.

The TSA signal is normally dominated by gear-meshing frequency components, which are masking much weaker vibration transients, that appear in signal due to developing gears faults. As the meshing frequency components are of a high energy, a common practice is their removal from the TSA signal. That removal leads to estimation of the so-called the classical residual signal (CRS) and can be undertaken in several ways. In this research, the CRS *r*(*t*) has been obtained by the method, described in [49], that is subtracting the average meshing teeth vibration signal from the TSA signal, according to the following Equation:

(6) $$r\;\left(t\right)\;=\;m\;\left(t\right)\;-\;\frac{1}{N_{t}}\overset{N_{t}-1}{\underset{k=0}{\sum }}m\;\left(t\;-\;kT_{m}\right)$$

where *m*(*t*) is the TSA signal, *T_m_* is the mesh period and *N_t_* is the number of pinion teeth.

The CRS have been used for WSC estimation.

**Figure 8:**
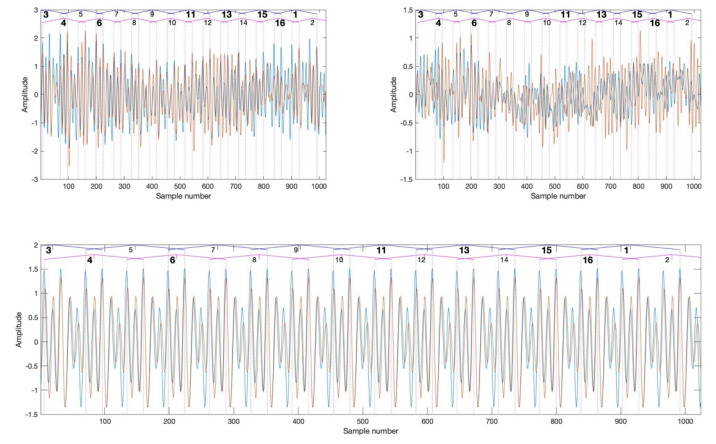
The time synchronous averaging (TSA) signal (**top left**), the classical residual signal (CRS) (**top right**) and the meshing components (**bottom**). Blue curves correspond to signal, representing the undamaged case, red curves correspond to signal, representing the damaged case.

Figure 8 presents the TSA signal and the CRS, recorded at the beginning (blue) and at the end of the test (red). In the bottom of Figure 8, the meshing components of the vibration signals, corresponding to undamaged (blue) and damaged (red) cases, are displayed. The meshing signal components that are removed from the TSA signal in order to obtain the CRS, have been used to align the vertical lines marking the tooth contact angle range limits with the actual angular positions, in which teeth of the gear and the pinion were in contact. A good match between width of the angular ranges and spacing between the meshing signal peaks is visible.

#### 3.2.2. Selection of Diagnostically Relevant Frequency Bands for the Proposed Technologies

Estimation of the WSC requires a choice of complex wavelet-type and optimal parameters of the wavelet transform. In the present research, the selected complex mother wavelet function is the Morlet mother wavelet function, that is expressed by Equation:

(7) $$\Psi \;\left(t^{\prime }\right)\;=\;\frac{1}{\sqrt{\pi f_{b}}}\;\left(e^{-i2\pi f_{c}t^{\prime }}\;-\;e^{-f_{b}{\left(\pi f_{c}\right)}^{2}}\right)\;e^{-{t^{\prime }}^{2}/f_{b}}$$

where *f_c_* is the central frequency of the mother wavelet and *f_b_* is the bandwidth parameter.

The bandwidth parameter defines a balance between the time and the frequency resolutions of the wavelet transform.

There are many different families of wavelet functions for various engineering applications. As the proposed wavelet cross-correlations employ both amplitude and phase information, this requires a choice of a complex wavelet type [50]. The main principle of a wavelet function selection for fault diagnosis is that a selected function should have a shape, similar to a shape of a signal, generated by a mechanical fault. In the present work, the Morlet mother wavelet function is selected due to the following main reasons: (i) there is a shape similarity between the decaying component of the Morlet wavelet and the decaying component of typical impulses, generated by gearbox and bearing local faults [51,52,53]; (ii) the Morlet wavelet is employing the Gaussian window and, therefore, is providing a highly efficient balance between time and frequency resolutions [54]; (iii) the Morlet wavelet is not a sharp edge function, and, therefore, it minimizes the spectral leakage [54,55]; (iv) the Morlet wavelet allows selection of highly efficient balance between time and frequency resolutions by two parameters: the centre frequency and the bandwidth parameter; (v) the wavelet transform with the Morlet wavelet is computationally efficient due to its link to the fast Fourier transform. Based on these advantages, the Morlet wavelet is well adapted for local fault detection in gearboxes [23,56,57,58,59].

It is known that the wavelet transform provides a good frequency resolution and a poor time resolution for low frequency signal components and a poor frequency resolution and a good time resolution for high-frequency signal components. In cases where transient vibration of a certain duration excites a gearbox in a broad frequency band, finding a compromise in setting *f_b_* parameter (Equation (7)), that would ensure optimal time-frequency resolution, for both low- and high-frequency resolution parameters may be difficult.

Therefore, *f_b_* parameter is calculated separately for each frequency in order to ensure that the duration of the wavelet matches the duration of vibration transients, generated by the gear’s local fault. Parameter *f_b_* was calculated for every frequency in such a way, that time-width of the wavelet at −3 dB level was corresponding to 1/15 of the period of the pinion rotation. Such an approach is proposed in [60] in order to match the time length of the wavelet with the duration of the vibration transient caused by the local gear fault.

**Figure 9:**
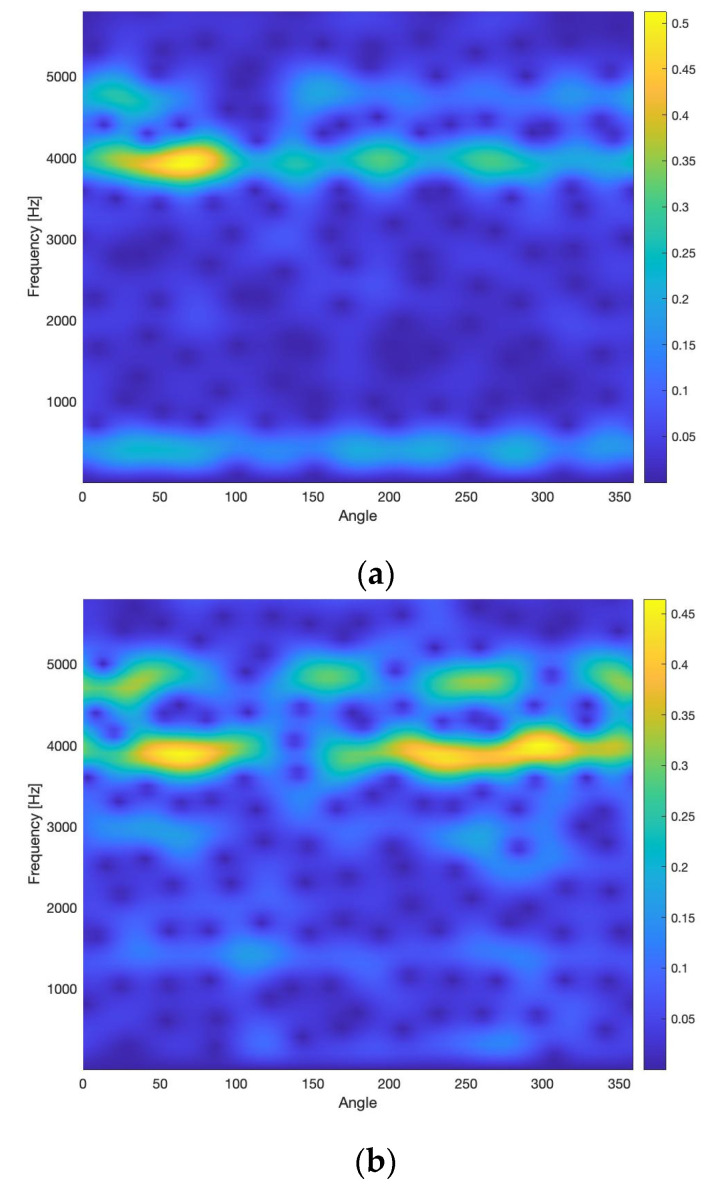
The wavelets scalograms of the vibration signal of the undamaged (**a**) and the damaged gearboxes (**b**).

The wavelet transform of the CRS (Figure 9), which is the base for estimation of the WSC3 and the WSC4, is estimated for frequencies, corresponding to the even orders of the pinion rotation speed. This was done to optimise calculation time. A further step towards calculation time optimisation could be usage of computationally efficient discrete wavelet transform, that will require selection of the adequate level of decomposition. In the presented research, the continuous wavelet transform is used.

**Figure 10:**
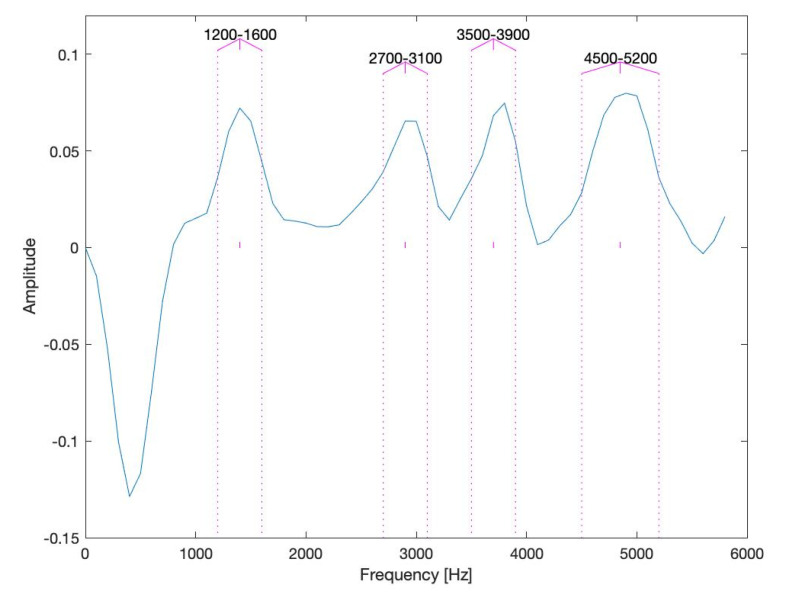
The absolute value of a difference between the wavelets scalograms of the vibration signals of the undamaged and the damaged gearboxes. Vertical lines mark the frequency bands that are considered as diagnostically relevant.

The proposed method of selection of diagnostically relevant frequency bands is carried out, based on analysis of the modulus of a difference between the wavelet scalograms, estimated for the CRSs, recorded between (0–10 × 10^6^) cycles (the undamaged conditions) and between (40 × 10^6^–50 × 10^6^) cycles (the damaged conditions). The amplitude vs. frequency plot, shown in Figure 10, indicates the frequency bands: <1200 Hz, 1600 Hz>; <2700 Hz, 3100 Hz>; <3500 Hz, 3900 Hz>; <4500 Hz, 5200 Hz>, in which the increase of vibration energy between the wavelet scalograms for the damaged and the undamaged cases are the most significant. Frequency components contained in these bands were used for estimation of WSC3 and WSC4 integrated features IWSC3 and IWSC4. This novel method of selection (identification) of diagnostically relevant frequency bands minimizes WSC feature processing time by narrowing it to identified frequency components, which is an essential step forward enabling industrial applications. 

The frequency bands, chosen for the integration on the IWSC3 and the IWSC4 features, are associated with mechanical properties of the gearbox under test. Although these relations are difficult to assess and such an assessment should be a subject of a specific relatively complicated modelling via model-driven diagnostic research, e.g., [61,62]. This assessment would also require an estimation of a transfer function between a gear’s meshing teeth and sensor location under operating load conditions. That would require complex experimental measurements, which would be difficult to carry out even in laboratory conditions, and an advanced numerical simulation, which would need to be validated. 

A large number of research works related to gearbox fault diagnosis via detection of fault related impacts use the data-driven approach [63]. In these works, selection of the diagnostically relevant frequency bands is normally performed via the spectral kurtosis, applied to vibration data, without model-based knowledge of dependencies between these frequency bands and mechanical properties of the gearboxes [49]. The data-driven approach used in this research, proposes selection of the relevant frequency bands taking into the account changes in the frequency content of signals recorded for the damaged and the undamaged cases. These signals are influenced by mechanical properties of the gearbox, therefore, the selected frequency bands are associated with mechanical properties of the test setup, although the exact analytical or modelling characters of this association remain unknown. 

Data- driven selection of relevant frequency bands, based only on the signal analysis, takes into the account mechanical properties of the gearbox and also the changes in the signal, determined by developing gear faults. One of the novelties of the presented research is a novel data-driven method for selection of diagnostically relevant frequency bands. Due to relative simplicity and effectiveness of the proposed method, it is an enhancement and an essential step towards industrialisation of the considered WSC-based technologies.

#### 3.2.3. Estimation of the Higher-Order Wavelet Spectral Cross-Correlation and the Integrated Wavelet Spectral Cross-Correlation (IWSC) Feature

The WSC of 3rd and 4th orders are estimated according to Equation (1), using the same parameters of digital signal processing. The averaging, represented by operator E (Equation (1)), is carried out for 30 consecutive complex wavelet transforms of the CRS signals. 

The integrated feature IWSC3 of the WSC of the 3rd order has been estimated for all possible triple combinations of spectral components; each spectral component belongs to one of four frequency bands, selected as diagnostically relevant (Figure 10). The integrated feature IWSC4 of the WSC of the 4th order has been estimated for all possible quadruple combinations of spectral components; each spectral component belongs to one of four frequency bands, selected as diagnostically relevant (Figure 10).

**Figure 11:**
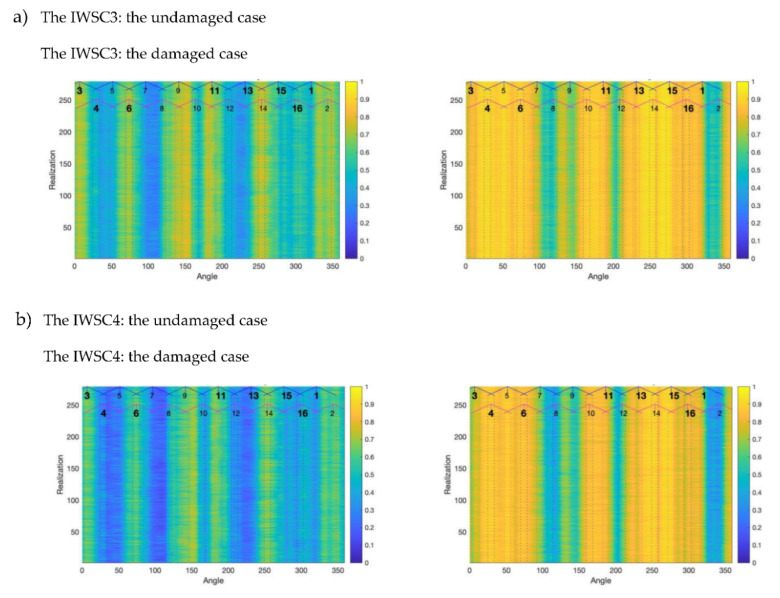
The integrated features IWSC3 (**a**) and IWSC4 (**b**). Vertical dotted lines mark the angular ranges, in which pinion teeth were in contact with gear teeth.

The plots in Figure 11 are related to 270 realisations of the IWSC3 (a) and the WSC4 (b) features, estimated for the same signals from the damaged and the undamaged gearboxes. The IWSC3 and the IWSC4 have been estimated respectively for 400 and 448 combinations of spectral components.

#### 3.2.4. Estimation of the Fisher Criterion

In spite of visible differences between the integrated features, obtained for the damaged and the undamaged gearboxes, visual evaluation of the integrated features does not allow identification of the damaged teeth. Therefore, IWSC3 and IWSC4 data sets, obtained for the damaged and the undamaged cases, have been compared by the Fisher criterion (FC), expressed by the following Equation [64]:

(8) $$\mathrm{FC}\;\left(\theta \right)\;=\;\frac{{\left(\mu_{D}\;\left(\theta \right)\;-\;\mu_{ND}\;\left(\theta \right)\;\right)}^{2}}{\sigma_{D}^{2}\;\left(\theta \right)\;+\;\sigma_{ND}^{2}\;\left(\theta \right)}$$

where *θ* denotes the angular position, for which the FC is estimated, *μ_D_* and *μ_ND_* are the mean values and *σ_D_* and *σ_ND_* are the standard deviation values of the integrated features, obtained for the damaged and the undamaged cases respectively.

As the IWSC3 and the IWSC4 are one-dimensional diagnostic features, their comparison has been carried out using the effective one-dimensional Fisher criterion, which is suitable and widely used for diagnostic effectiveness estimation for one-dimensional diagnostic features [64,65,66].

**Figure 12:**
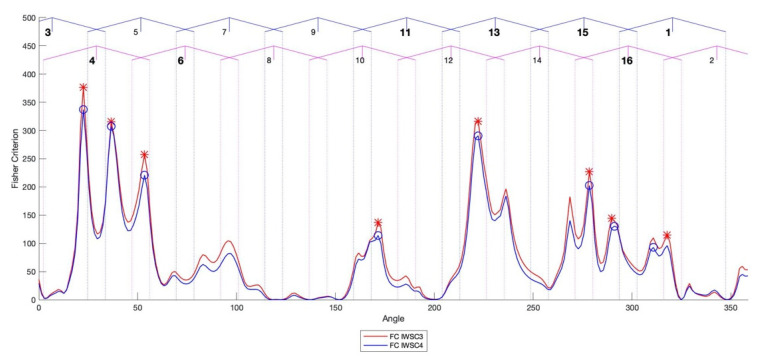
The Fisher criteria (FC) for the IWSC3 (blue) and the IWSC4 (red). Bold numbers in the top of the plot mark positions of the teeth, on which local faults were observed. Local extreme points have been marked with circles (for the IWSC3) and stars (for the IWSC4).

The FC tends to give higher values for diagnostic features, featuring not only significant difference in mean value, but also low variances and, therefore, identifies the angular positions of the pinion on which the changes of the integrated features are significant between the damaged and undamaged case and also stable for number of integrated features estimations. In the cases of the IWSC3 and the IWSC4, FC local maxima explicitly correlate with angular locations of the damaged teeth. Figure 12 shows comparison between the Fisher criteria of the IWSC3 and the IWSC4.

## 4. Analysis of the Results

High values of the Fisher criterion, which allow effective gearbox fault diagnosis and localisation of individual teeth, affected by micro-pitting, prove the high validity of the WSC-based diagnosis. In order to quantify the diagnostic effectiveness of the WSC, an additional analysis of overall diagnosis effectiveness and the total probability of correct diagnosis has been carried out.

**Figure 13:**
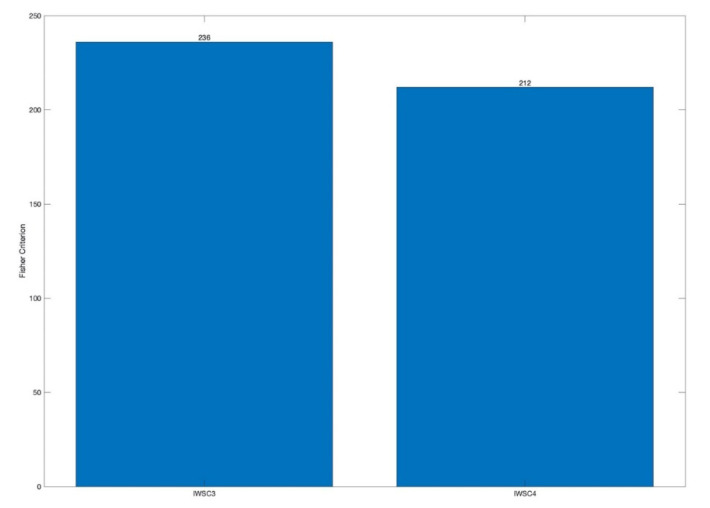
Bar plot, showing the averaged values of the FC local maxima, estimated for the IWSC3 and the IWSC4.

FC local maxima are good indicators of diagnosed fault locations. The higher FC value, the higher validity of indication. In order to evaluate effectiveness of the IWSC3 and the IWSC4 in a generic way, i.e., without breakdown to individual teeth, the values of FC local maxima have been averaged in order to quantify the effectiveness of the damaged teeth diagnosis as one collective statistic. Figure 13 present the average FC values, obtained for the IWSC3 and the IWSC4.

High averaged values of the FC local maxima, obtained for the IWSC3 and the IWSC4, prove high diagnosis effectiveness of the proposed technologies.

To further quantify the diagnostic effectiveness of the WSC, the estimates of the total probability of correct diagnosis (TPCD) has been also evaluated. Estimation has been carried out for the WSC3 and the WSC4 technologies.

The TPCD has been estimated using the following formula:

(9) $$\mathrm{TPCD}\;=\;\frac{t_{p}\;+\;t_{n}}{2}$$

where *t_p_*, *t_n_* are respectively the estimates of the probabilities of true positive and true negative diagnosis.

**Figure 14:**
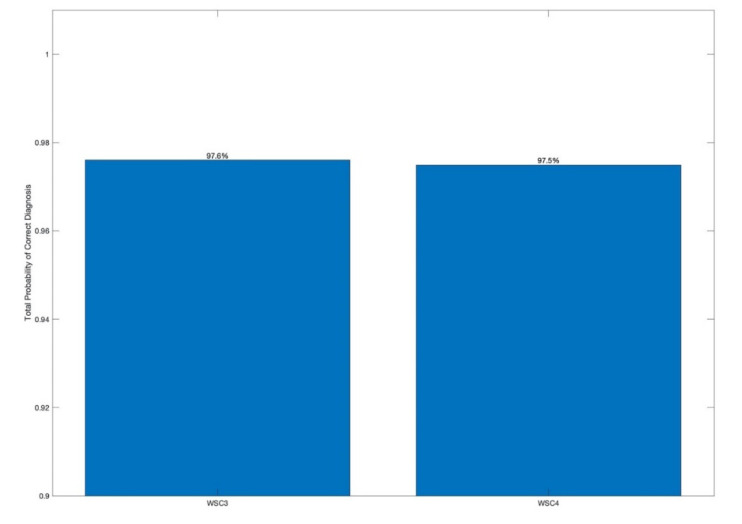
Bar plot, showing the estimates of the total probabilities of correct diagnosis, evaluated for the IWSC3 and the IWSC4.

The estimates of the TPCD have been evaluated based on 6080 integrated feature values for each technology, and are shown in Figure 14. For diagnostic decision-making for each one-dimensional diagnostic feature, the IWSC3 and the IWSC4, a threshold-based decision-making rule is used by employing the Bayesian criterion [64].

The estimates of the TPCD for the IWSC3 and the IWSC4 are 97.6% and 97.5%. The TPCD estimation was based on a comprehensive data set, consisting of 6080 diagnostic features, that are based on 1.14 million raw signal sections, corresponding to a single pinion revolution. That statistical data correspond to 6 h and 20 min of recorded vibration signal. Thus, the validity of the experimental results has been proven by significant amounts of vibration data. The high level of validity of the obtained results has been proven not only by analysis of significant sets of data, but also by comparison of the obtained vibration diagnosis results with results of accurate gearbox diagnosis, carried out by the independent stereo optical method during experimental trials, as described in Section 3.1.

It can be seen from the obtained results (Figure 13 and Figure 14) that both proposed spectral cross-correlation technologies, the IWSC3 and the IWSC4, provide comparable diagnosis effectiveness for local gearbox damage.

For novel experimental comparison of gearbox fault diagnosis effectiveness for the proposed technologies and the HOS technologies, the wavelet bicoherence and the wavelet tricoherence [23] are used, that are the HOS techniques of order 3 and 4 respectively. For comparison purposes, the integrated WSC features, the integrated wavelet bicoherence features, and the integrated wavelet tricoherence features are estimated for the same gearbox vibration signals.

Based on the processing of experimental vibration data, the estimates of the total probabilities of incorrect diagnosis of gearbox damage provided by the wavelet bicoherence technology and the wavelet tricoherence technology are, respectively, 4.7% and 26.2%.

The estimates of the total probabilities of incorrect diagnosis are 2.4% for the IWSC3 and 2.5% for the IWSC4. Comparing the corresponding estimates of the total probabilities of incorrect diagnosis, the difference in diagnostic effectiveness between the compared technologies becomes more explicit.

The effectiveness gain in the total probability of incorrect gearbox damage diagnosis for the IWSC3 technology, that is a ratio of the estimates of the total probability of incorrect gearbox damage diagnosis by the bicoherence technology and the IWSC3 technology, is 1.96. The effectiveness gain in the total probability of incorrect gearbox damage diagnosis for the IWSC4 technology, that is a ratio of the estimates of the total probability of incorrect gearbox damage diagnosis by the tricoherence technology and the IWSC4 technology, is 10.5. The wavelet bicoherence technology detects all eight damaged teeth; the wavelet tricoherence technology detects six damaged teeth out of eight.

The diagnosis effectiveness estimations by the Fisher criterion are as follows: the averaged Fisher criteria for the WSC3 and the WSC4 technologies are 236 and 212, respectively, the averaged Fisher criteria for the wavelet bicoherence and the wavelet tricoherence technologies are 173 and 2, respectively.

The higher diagnostic effectiveness of the WSC technologies, compared with the HOS technologies, finds its reflection in comparison of the TPCDs and the averaged FCs.

A very serious limitation of the wavelet HOS is the constraint, related to selection of analysed frequencies: i.e., in the case of 3rd-order bispectrum, the highest analysed frequency must equal the sum of two lower frequencies, and in the case of 4th order trispectrum, the highest analysed frequency must equal the sum of three lower frequencies. The frequency bands, chosen as diagnostically relevant, are not always fulfil the requirements of the HOS definition; an example is in Figure 10. This limitation usually has a very negative influence on quality of fault diagnosis and limits fault types, that could be effectively diagnosed by the wavelet HOS.

The proposed WSC technologies allow analysis of any combination between freely chosen frequency components; therefore, these technologies significantly extend capabilities of the wavelet HOS. As shown here, the proposed WCS technologies are also more effective, than the wavelet HOS, for early gear fault diagnosis. One of the important achievements of the proposed research is obtaining a high level of diagnosis reliability of naturally developed gear micro-pitting at a very early stage.

## 5. Conclusions

(1). The novel vibration sensor-based diagnosis technologies, built on the higher order wavelet spectral cross-correlation (WSC), are proposed, investigated and applied to gearbox vibration health monitoring for the first time in worldwide terms. The physical sense of the proposed technologies is that the complex spectral components, that are appearing in vibration data due to gearbox local damage, exhibit non-zero spectral cross-correlations.

(2). Gearbox vibration sensor-based data from a laboratory test rig, consisting of two back-to-back industrial gearboxes were captured by a vibration sensor, the charge-type linear accelerometer, and are employed for novel experimental investigation of the proposed technologies. Vibration data were captured for two gearbox conditions: without damage and with the naturally produced early stage of gearbox local damage (a micro-pitting). Analysed gear vibration signals were recorded during an endurance gearbox test, in course of which a natural micro-pitting has developed, but reached only a very early stage, affecting from 0.3% to 0.7% of tooth flank surface, and was observed on eight out of 16 teeth of the pinion. A randomly selected signal section, recorded at the beginning of the endurance test, represents an undamaged case, and randomly selected signal section, recorded at the end of the endurance test, represents the damaged case.

(3). The novel experimental investigation of the proposed diagnosis technologies, applied to gearbox vibration diagnosis, has shown a distinctive difference between the proposed integrated diagnostic features for undamaged and damaged cases: the diagnostic features essentially increase, if gearbox damage is present. A novel experimental investigation of the technologies has also shown a high effectiveness of the proposed diagnostic technologies for gearbox local tooth damage diagnostics, which has been proven by the comprehensive high validity experimental trials.

The IWSC3 and the IWSC4 integrated diagnostic features, estimated for signals from the damaged and undamaged gearboxes, have been compared by the Fisher criterion (FC). Angular positions of FC local maxima are correlated with angular ranges, in which damaged teeth of the pinion were in contact with teeth of gear. All damaged pinion teeth were successfully diagnosed by the proposed technologies.

The estimates of the total probability of correct diagnosis (TPCD), evaluated for the proposed technologies, are based on a set of 6080 diagnostic features. Estimates of the TPCD, obtained for the WSC3 and the WSC4, were respectively 97.6% and 97.5%. Thus, estimates of the total probability of incorrect diagnosis was 2.4% for the WSC3 and 2.5% for the WSC4. These experimental effectiveness estimates confirm successful gearbox fault diagnosis by the proposed technologies at a very early stage of gear local tooth damage.

(4). The novel method of identification of diagnostically important frequency bands is proposed and applied for the WSC in order to minimize WSC feature-processing time by narrowing it to selected frequency components, enabling industrial applications. Method realisation involved estimation of the gearbox residual signals and analysis of the difference between the wavelet scalograms of the residual signals for the undamaged and the damaged cases. The proposed method allowed us to define four diagnostically important frequency bands for the diagnosed gearbox vibration data.

(5). The novel experimental comparisons of the proposed technologies and the HOS technologies were performed based on the same gearbox vibration data.

The effectiveness gain in the total probability of incorrect gearbox damage diagnosis was 1.95 for comparison of the WSC3 and the wavelet bicoherence 10.5 for comparison of the WSC4 and the wavelet tricoherence

The differences in diagnostic effectiveness criterion, the averaged Fisher criterion, were as follows: the averaged Fisher criteria for the WSC3 and the WSC4 were 236 and 212, respectively, the averaged Fisher criteria for the wavelet bicoherence and the wavelet tricoherence were 173 and 2 respectively.

Thus, the comparisons clearly show the higher gearbox damage diagnosis effectiveness by the proposed technologies over the HOS technologies.

(6). The novel experimental comparison of the proposed spectral cross-correlation technologies of order 3 and 4 are performed, using the total probability of incorrect diagnosis and the Fisher criterion. The results obtained indicate that both proposed spectral correlation technologies, the IWSC3 and the IWSC4, provide comparable diagnosis effectiveness for local gearbox damage.

(7). An important advantage of the proposed technologies over the HOS technologies is an unconstrained choice of frequency components, between which the higher order WSC is estimated. This advantage allows focusing on all frequency bands that contain diagnostically important frequency components and, thus, is not limited damage types, that could be effectively diagnosed by the proposed technologies.

It is clear that the WSC technologies are superior over the wavelet HOS technologies, used so far, and are a significant step towards early-stage fault diagnosis.

The proposed novel diagnosis technologies, the higher-order spectral cross-correlation technologies, presents a new concept and will make a major influence on vibration damage diagnostics for various complex electromechanical systems and also can be effectively employed for other damage-diagnosis technologies: e.g., low-frequency acoustic damage diagnosis technologies, ultrasound damage diagnosis technologies, etc.

## References

[1] Collis W.B., White P.R., Hammond J.K. (1998). Higher-order spectra: The bispectrum and trispectrum. Mech. Syst. Signal Process..

[2] Fackrell J.W.A., White P.R., Hammond J.K., Pinnington R.J., Parsons A.T. (1995). The interpretation of the bispectra of vibration signals I. theory. Mech. Syst. Signal Process..

[3] Fackrell J.W.A., White P.R., Hammond J.K., Pinnington R.J., Parsons A.T. (1995). The interpretation of the bispectra of vibration signals—II. Experimental results and applications. Mech. Syst. Signal Process..

[4] Gelman L., Kırlangıç A.S. (2020). Novel vibration structural health monitoring technology for deep foundation piles by non-stationary higher order frequency response function. Struct. Control Heal. Monit..

[5] Kiciński W., Szczepański A. (2004). Quadratic phase coupling phenomenon and its properties. Hydroacoustics.

[6] Gelman L., Petrunin I., Parrish C., Walters M. (2020). Novel health monitoring technology for in-service diagnostics of intake separation in aircraft engines. Struct. Control Heal. Monit..

[7] Urresty J.C., Riba J.R., Romeral L. (2013). Diagnosis of interturn faults in PMSMs operating under nonstationary conditions by applying order tracking filtering. IEEE Trans. Power Electron..

[8] Ruiz J.R., Garcia A., Romeral L., Cusidó J. (2010). Demagnetization diagnosis in permanent magnet synchronous motors under non-stationary speed conditions. Electr. Power Syst. Res..

[9] Randall R.B. (2016). Vibration-based diagnostics of gearboxes under variable speed and load conditions. Meccanica.

[10] Plöger D.F., Zech P., Rinderknecht S. (2019). Vibration signature analysis of commodity planetary gearboxes. Mech. Syst. Signal Process..

[11] Sawalhi N., Randall R.B. (2014). Gear parameter identification in a wind turbine gearbox using vibration signals. Mech. Syst. Signal Process..

[12] Huang N.E., Shen Z., Long S.R., Wu M.C., Snin H.H., Zheng Q., Yen N.C., Tung C.C., Liu H.H. (1998). The empirical mode decomposition and the Hubert spectrum for nonlinear and non-stationary time series analysis. Proc. R. Soc. A Math. Phys. Eng. Sci..

[13] Wu D., Wang J., Wang H., Liu H., Lai L., He T., Xie T. (2020). An automatic bearing fault diagnosis method based on characteristics frequency ratio. Sensors.

[14] Zhao L., Yu W., Yan R. (2016). Gearbox Fault Diagnosis Using Complementary Ensemble Empirical Mode Decomposition and Permutation Entropy. Shock Vib..

[15] Wang Z., Wang J., Duv W. (2018). Research on fault diagnosis of gearbox with improved variational mode decomposition. Sensors.

[16] Delprete C., Brusa E., Rosso C., Bruzzone F. (2020). Bearing Health Monitoring Based on the Orthogonal Empirical Mode Decomposition. Shock Vib..

[17] Rivola A. Comparison between second and higher order spectral analysis in detecting structural damages. Proceedings of the Seventh International Conference on Recent Advances in Structural Dynamics.

[18] Rivola A., White P.R. (1998). Bispectral analysis of the bilinear oscillator with application to the detection of fatigue cracks. J. Sound Vib..

[19] Park H. (2008). Nonlinearity Detection for Condition Monitoring Utilizing Higher-order Spectral Analysis Diagnostics. Ph.D. Thesis.

[20] Chua K.C., Chandran V., Acharya U.R., Lim C.M. (2011). Application of higher order spectra to identify epileptic EEG. J. Med. Syst..

[21] Hinich M.J. (1990). Detecting a Transient Signal by Bispectral Analysis. IEEE Trans. Acoust..

[22] Van Milligen B.P., Sanchez E., Estrada T., Hidalgo C., Brañas B., Carreras B., García L. (1995). Wavelet bicoherence: A new turbulence analysis tool. Phys. Plasmas.

[23] Combet F., Gelman L., Lapayne G. (2012). Novel detection of local tooth damage in gears by the wavelet bicoherence. Mech. Syst. Signal Process..

[24] Li Y., Wang X., Lin J. Fault diagnosis of rolling element bearing using nonlinear wavelet bicoherence features. Proceedings of the 2014 International Conference on Prognostics and Health Management.

[25] Gelman L., Murray B., Patel T.H.H., Thomson A. (2014). Vibration diagnostics of rolling bearings by novel nonlinear non-stationary wavelet bicoherence technology. Eng. Struct..

[26] Gelman L., Murray B., Patel T.H., Thomson A. (2013). Diagnosis of bearings by novel non-linear non-stationary higher order spectra. Insight Non-Destructive Test. Cond. Monit..

[27] Gelman L., Solinski K., Shaw B., Vaidhianathasamy M. (2017). Vibration diagnosis of a gearbox by wavelet bicoherence technology. Insight Non-Destructive Test. Cond. Monit..

[28] Gelman L., Parrish C., Petrunin I., Walters M. (2017). Novel in-service combustion instability detection using the chirp Fourier higher order spectra. Int. J. Progn. Heal. Manag..

[29] Gryllias K.C., Gelman L., Shaw B., Vaidhianathasamy M. (2010). Local damage diagnosis in gearboxes using novel wavelet technology. Insight-Non-Destructive Test. Cond. Monit..

[30] McCormick A.C., Nandi A.K. (1999). Bispectral and trispectral features for machine condition diagnosis. IEE Proc. Vis. Image Signal Process..

[31] Yan X., Jia M. (2019). Application of CSA-VMD and optimal scale morphological slice bispectrum in enhancing outer race fault detection of rolling element bearings. Mech. Syst. Signal Process..

[32] Mao Y., Jia M., Yan X. (2020). A new bearing weak fault diagnosis method based on improved singular spectrum decomposition and frequency-weighted energy slice bispectrum. Meas. J. Int. Meas. Confed..

[33] Wang F., Fang L. (2019). Bispectrum Texture Feature Manifold for Feature Extraction in Rolling Bear Fault Diagnosis. Math. Probl. Eng..

[34] Gelman L., Murray B., Patel T.H., Thomson A. (2013). Novel decision-making technique for damage diagnosis. Insight-Non-Destructive Test. Cond. Monit..

[35] Du X. (2019). Fault detection using bispectral features and one-class classifiers. J. Process Control.

[36] Parker B.E., Ware H.A.A., Wipf D.P.P., Tompkins W.R.R., Clark B.R.R., Larson E.C.C., Poor H.V. (2000). Fault diagnostics using statistical change detection in the bispectral domain. Mech. Syst. Signal Process..

[37] Li Z., Yan X., Yuan C., Zhao J., Peng Z. (2011). Fault detection and diagnosis of a gearbox in marine propulsion systems using bispectrum analysis and artificial neural networks. J. Mar. Sci. Appl..

[38] Guoji S., McLaughlin S., Yongcheng X., White P. (2014). Theoretical and experimental analysis of bispectrum of vibration signals for fault diagnosis of gears. Mech. Syst. Signal Process..

[39] Guo J., Zhen D., Li H., Shi Z., Gu F., Ball A.D. (2020). Fault detection for planetary gearbox based on an enhanced average filter and modulation signal bispectrum analysis. ISA Trans..

[40] Gelman L., White P., Hammond J. (2005). Fatigue crack diagnostics: A comparison of the use of the complex bicoherence and its magnitude. Mech. Syst. Signal Process..

[41] Gelman L., Petrunin I. (2007). The new multidimensional time/multi-frequency transform for higher order spectral analysis. Multidimens. Syst. Signal Process..

[42] Gelman L., Patel T.H., Murray B., Thomson A. (2013). Rolling Bearing Diagnosis Based on the Higher Order Spectra. Int. J. Progn. Heal. Manag..

[43] Gelman L., Ottley M. (2006). New processing techniques for transient signals with non-linear variation of the instantaneous frequency in time. Mech. Syst. Signal Process..

[44] Gelman L. (2007). Adaptive time-frequency transform for non-stationary signals with nonlinear polynomial frequency variation. Mech. Syst. Signal Process..

[45] Gelman L., Gould J.D. (2007). Time-frequency chirp-Wigner transform for signals with any nonlinear polynomial time varying instantaneous frequency. Mech. Syst. Signal Process..

[46] Zhang R., Gu X., Gu F., Wang T., Ball A.D. (2017). Gear wear process monitoring using a sideband estimator based on modulation signal bispectrum. Appl. Sci..

[47] Guo J., Shi Z., Li H., Zhen D., Gu F., Ball A.D. (2018). Early fault diagnosis for planetary gearbox based wavelet packet energy and modulation signal bispectrum analysis. Sensors.

[48] Gelman L., Patel T.H., Persin G., Murray B., Thomson A. (2013). Novel Technology Based on the Spectral Kurtosis and Wavelet Transform for Rolling Bearing Diagnosis. Int. J. Progn. Heal. Manag..

[49] Combet F., Gelman L. (2009). Optimal filtering of gear signals for early damage detection based on the spectral kurtosis. Mech. Syst. Signal Process..

[50] Yang S., Gu X., Liu Y., Hao R., Li S. (2020). A general multi-objective optimized wavelet filter and its applications in fault diagnosis of wheelset bearings. Mech. Syst. Signal Process..

[51] Nikolaou N.G., Antoniadis I.A. (2002). Demodulation of Vibration Signals Generated By Defects in Rolling Element Bearings Using Complex Shifted Morlet Wavelets. Mech. Syst. Signal Process..

[52] Wang X.Y., Makis V., Yang M. (2010). A wavelet approach to fault diagnosis of a gearbox under varying load conditions. J. Sound Vib..

[53] Elbarghathi F., Tran V.T., Gu F., Ball A. Multi-stages helical gearbox fault detection using vibration signal and Morlet wavelet transform adapted by information. Proceedings of the COMADEM 2013.

[54] Gryllias K.C., Antoniadis I.A. (2013). Estimation of the instantaneous rotation speed using complex shifted Morlet wavelets. Mech. Syst. Signal Process..

[55] Cohen M.X. (2019). A better way to define and describe Morlet wavelets for time-frequency analysis. Neuroimage.

[56] Staszewski W.J., Tomlinson G.R. (1994). Application of the wavelet transform to fault detection in a spur gear. Mech. Syst. Signal Process..

[57] Wang W.J., McFadden P.D. (1996). Application of wavelets to gearbox vibration signals for fault detection. J. Sound Vib..

[58] Wang W.Q., Ismail F., Farid Golnaraghi M. (2001). Assessment of Gear Damage Monitoring Techniques Using Vibration Measurements. Mech. Syst. Signal Process..

[59] Gelman L., Harish Chandra N., Kurosz R., Pellicano F., Barbieri M., Zippo A. (2016). Novel spectral kurtosis technology for adaptive vibration condition monitoring of multi-stage gearboxes. Insight Non-Destructive Test. Cond. Monit..

[60] Gelman L., Petrunin I., Jennions I.K., Walters M. (2012). Diagnostics of Local Tooth Damage in Gears by the Wavelet Technology. Int. J. Progn. Heal..

[61] Dadon I., Koren N., Klein R., Lipsett M.G., Bortman J. (2020). Impact of gear tooth surface quality on detection of local faults. Eng. Fail. Anal..

[62] Mączak J., Jasiński M. (2018). Model-based detection of local defects in gears. Arch. Appl. Mech..

[63] Djeziri M.A., Benmoussa S., Zio E., Sayed-Mouchaweh M. (2020). Review on Health Indices Extraction and Trend Modeling for Remaining Useful Life Estimation. Artificial Intelligence Techniques for a Scalable Energy Transition.

[64] Webb A.R. (2002). Statistical Pattern Recognition.

[65] Huang Y., Liu C., Zha X.F., Li Y. (2010). A lean model for performance assessment of machinery using second generation wavelet packet transform and Fisher criterion. Expert Syst. Appl..

[66] Hu Q., Si X.S., Zhang Q.H., Qin A.S. (2020). A rotating machinery fault diagnosis method based on multi-scale dimensionless indicators and random forests. Mech. Syst. Signal Process..

